# The shifting shelf task: a new, non-verbal measure for attentional set shifting

**DOI:** 10.1098/rspb.2022.1496

**Published:** 2023-01-25

**Authors:** E. Reindl, C. J. Völter, Z. Civelek, L. Duncan, Z. Lugosi, E. Felsche, E. Herrmann, J. Call, A. M. Seed

**Affiliations:** ^1^ School of Psychology and Neuroscience, University of St Andrews, St Andrews KY16 9JP, UK; ^2^ Department of Anthropology, Durham University, Durham DH1 3LE, UK; ^3^ Messerli Research Institute, University of Veterinary Medicine Vienna Medical University of Vienna, University of Vienna, Vienna 1210, Austria; ^4^ School of Medicine, Medical Sciences and Nutrition, University of Aberdeen, Aberdeen AB24 3FX, UK; ^5^ Division of Psychology, University of Stirling, Stirling FK9 4LA, UK; ^6^ Faculty of Social Sciences and Public Policy, School of Education, Communication and Society, King's College London, London WC2R 2LS, UK; ^7^ Department of Comparative Cultural Psychology, Max Planck Institute for Evolutionary Anthropology, Leipzig 04103, Germany; ^8^ Department of Psychology, University of Portsmouth, Portsmouth PO1 2UP, UK

**Keywords:** attentional set shifting, cognitive flexibility, executive functions, rule switching, comparative cognition

## Abstract

Attentional set shifting is a core ingredient of cognition, allowing for fast adaptation to changes in the environment. How this skill compares between humans and other primates is not well known. We examined performance of 3- to 5-year-old children and chimpanzees on a new attentional set shifting task. We presented participants with two shelves holding the same set of four boxes. To choose the correct box on each shelf, one has to switch attention depending on which shelf one is currently presented with. Experiment 1 (forty-six 3- to 5-year olds, predominantly European White) established content validity, showing that the majority of errors were specific switching mistakes indicating failure to shift attention. Experiment 2 (one hundred and seventy-eight 3- to 6-year olds, predominantly European White) showed that older children made fewer mistakes, but if mistakes were made, a larger proportion were switching mistakes rather than ‘random’ errors. Experiment 3 (52 chimpanzees) established suitability of the task for non-human great apes and showed that chimpanzees' performance was comparable to the performance of 3- and 4-year olds, but worse than 5-year olds. These results suggest that chimpanzees and young children share attentional set shifting capacities, but that there are unique changes in the human lineage from 5 years of age.

## 1. Introduction

Executive functions (EFs) describe the range of cognitive processes underpinning complex goal-directed behaviour and higher cognitive functions such as mental time travel or theory of mind [1,2]. A core part of EF is *attentional set shifting*: the ability to quickly switch attention between different rules, instructions or ‘mental sets’, allowing individuals to adjust to changing environments [2–7]. An *attentional set* results from a bias to direct one's attention preferentially to one stimulus or group of stimuli over others, leading to heightened processing, with inhibited processing of other stimuli [4]. This processing bias typically results from the individual's learning history.

In humans, attentional set shifting capacities grow substantially during childhood and well into adolescence and early adulthood [8]. Attentional set shifting skills in early childhood are predictive of later academic and professional success, health, wealth and relationship quality, suggesting that supporting the development of EF in early years can have beneficial outcomes later in life [1,6,8–15]. Therefore, research has aimed to increase our understanding of the structure and development of EF and their underlying neural processes. For example, the Dynamic Field Theory aims to explain the neural processes underlying attentional set shifting and its development by simulating the neural dynamics when attempting different attentional set shifting tasks [16,17].

However, our understanding of attentional set shifting in early childhood is incomplete, which is partly owing to a shortage of measures suitable for young children [18]. Several studies using a latent variable approach to investigate the structure of EF in early childhood were not able to include attentional set shifting as a separate EF component as the researchers were not able to find enough tasks [19]. The current study aims to introduce a novel attentional set shifting task suitable for use with young children and non-human primates.

Investigating attentional set shifting in non-human primates can provide insights into the evolution of this ability and which aspects might be unique to modern humans [2,20–24]. Researchers have investigated different aspects of cognitive flexibility [3,4,23,25,26]. One line of research focuses more broadly on *behavioural* flexibility (i.e. the ability to change behaviour in response to environmental feedback; [26]), without a specific focus on the underlying cognitive mechanisms. These studies used problem-solving (e.g. tool use) tasks to investigate the ability to inhibit previously successful behaviours when they become inefficient and to switch to novel solutions (see reviews in [26,27]). They provide evidence for both flexibility and conservatism in non-human primates, with researchers only starting to examine potential factors responsible for the mixed results, such as social tolerance, persistence or task-specific features such as the degree of inefficiency of the initial solution [25,26]. The cognitive mechanisms underlying this innovative problem-solving remain largely unexplored, as does the relationship between behavioural flexibility and attentional set shifting.

Herrmann & Tomasello [21] investigated attention shifting in 4- and 5-year-old children, sub-adult and adult chimpanzees in a task based on foraging behaviour. While 4-year olds and chimpanzees performed similarly when having to monitor two identical devices and switch between them to collect a reward, both groups were outperformed by 5-year olds. In a more challenging experiment participants had to monitor two different tasks and shift their attention away from the continuously rewarding first task to the second which only occasionally released rewards. This proved to be equally challenging for all groups. A crucial difference to other tests of attentional set shifting (see below) is that participants had to switch their attention in the absence of any conflicting stimuli, making comparability to other studies more difficult and leaving unclear which processes might underpin the observed differences.

Another line of research involves the *reversal learning* paradigm, a forced switch task in which individuals learn to respond to one of two stimuli and—after having learned this—enter a second phase in which choosing the other stimulus is rewarded (e.g. [28]). There is evidence for differences in reversal learning capacities in the primate lineage, with great apes learning to switch their response more quickly than other non-human primates [29–31], and preschoolers outperforming monkeys and great apes [32,33]. However, reversal learning differs from attentional set shifting as it only requires learning a new stimulus-response association after a change in reward contingencies but no formation of and switching between mental sets or rules [2,3,23,25,34,35]. Additionally, there are differences in the brain areas involved [36]. For a complete perspective on attentional control processes we need to look at cases in which the role of shifting attention, rather than just changing behaviour, is examined.

Attentional set shifting has been assessed using forced switch and optional switch paradigms. Two commonly used forced switch tasks are the *Wisconsin card sort task* (WCST) for human adults [37] which has been adapted for non-human primates (*conceptual set shifting task* (CSST); [38]) and young children (dimensional change card sort (DCCS) [39,40]), and the *intradimensional/extradimensional shift (ID/ED)* task suitable for humans and non-human animals [4,18,35,41]. Here, participants use trial-and-error (an exception being the DCCS, in which there are verbal instructions) to learn which dimension to attend to. At the end of this phase, participants have often formed an attentional set (however, this is not necessary to solve the initial task; [3]). Then the rules change (unannounced, apart from in the DCCS), requiring the individual to overcome their attentional set (if they have formed one) and switch attention to the newly relevant dimension (only this new dimension is being rewarded, thus the label ‘forced switch’). While chimpanzees, similar to humans, experience an age-related decline in attentional set shifting in the CSST, even young adult chimpanzees require hundreds of trials more to reach the learning criterion compared to human adults doing the WCST [42]. Comparisons between young humans and non-human great apes on a card sorting test have not yet been carried out, possibly because the version for children, the DCCS, requires language, a feature that cannot easily be modified [2,23].

Using an optional switch paradigm, Pope *et al*. [43] showed that chimpanzees and baboons were more likely than adults to switch away from a learned inefficient, abstract rule to using a shortcut solution. This was not interpreted as non-human primates being more flexible than humans; instead, it was suggested that chimpanzees and baboons were less susceptible to forming a cognitive set in the initial learning phase because of differences in human and non-human sequence learning. Humans' enhanced rule learning abilities, working memory, verbal encoding and chunking abilities might have led them to process the sequences more holistically, making it more difficult to isolate shortcuts from the sequences. This study also highlights that in order to compare the ability to shift attention between species, the task should promote the formation of mental sets equally for all species, otherwise results that look like differences in flexibility might just reflect a lack of mental set formation, lower levels of learning or shorter memory for the previous solution.

Researchers have called for the development of additional attentional set shifting tasks because the DCCS and most other attentional set shifting tasks for children (e.g. the *preschool attentional switching task*, [44]; the *flexible induction of meaning* task, [45]; the *trail making test for preschoolers,* [46]; the *shape school,* [47] and the *object classification task for children,* [48]) rely on language [16,18]. Yet non-verbal tasks are needed to understand how young learners fare in tasks with unannounced rule changes and a lack of verbal instructions. They can also be used for comparative studies and can more easily be adapted for cross-cultural research. We designed an attentional set shifting task with minimal verbal demands and an implicit rule structure, suitable for various species, to allow for investigation and comparison of attentional set shifting between species.

In the *shifting shelf* task (electronic supplementary material, figure S1), participants are presented with two sets of shelves, each containing an upper and a lower shelf and holding a set of maximally four cups (two cups per shelf). In training, with changing left–right position of cups, participants learn which cup is consistently rewarded on each set of shelves. The rewarded cups differ between the sets, e.g. on the right set, the green cup on the bottom shelf is correct, on the left set, the pink cup on the top shelf is rewarded. In the test, both sets hold the same four cups (e.g. yellow, pink, green and orange). To choose the correct cup on each set participants need to shift their attention towards the correct cup based on the location (left–right) of the set they can currently choose from. If participants fail to switch attention, they will commit a specific type of mistake—a *switching mistake*, defined as choosing the cup that is rewarded on the other shelf—instead of choosing a cup that is never rewarded (‘random’ error). Switching mistakes are a signature of failed attention shifting (or perseveration).

We studied children aged 3 to 6 years from the UK and sanctuary-living chimpanzees of various ages. Choosing this age range for children had several reasons: the preschool period exhibits the fastest and most radical growth in EF (paralleling the maturation of the frontal lobes) and is of particular interest to developmental researchers [49]. Studying EF in children often allows us to more easily identify their role in other cognitive skills compared to when studying adults. Comparative developmental psychology has often found comparing non-human animals to preschoolers (as opposed to adults) to be a good starting point, as choosing a sample of young children can decrease (but of course not eliminate) the influence of formal schooling and many years of cultural experience on task performance. We already know that culture changes cognition substantially, letting adults outperform any other species on cognitive tasks, so comparisons with adults will tell us less about the evolutionary and developmental origins of EF.

Experiment 1 (E1) is a proof-of-concept study in which we establish internal validity of the task. To be considered an attentional set shifting task, we expected our task to reveal a specific error type indicative of a failure to switch attention [50]: switching mistakes. We hypothesized that if participants formed an attentional set towards the relevant cup on each shelf, the majority of errors in the test would be switching mistakes instead of choosing a cup that had never been rewarded. In experiment 2 (E2), we replicated E1 with a bigger sample and investigated potential age effects. In experiment 3 (E3), we investigated the performance of two groups of chimpanzees on the same task.

## 2. Experiment 1 (children)

### (a) Methods

#### (i) Participants

We tested 46 children between 3 years 2 months and 5 years 1 month (mean = 50.61, s.d. = 5.77 months, range = 38–61) in five nurseries in Scotland, UK, between January and March 2019. There were thirteen 3-year olds, thirty-two 4-year olds and one 5-year old. See the electronic supplementary material, for further information. Only children whose parents provided written informed consent were asked whether they would like to take part. Ethical approval for all experiments was granted by the University of St Andrews, UK, School of Psychology and Neuroscience Ethical Review Committee (approval code: PS13481).

### (b) Materials

We used two visually distinct sets of shelves, 12 cups, a cardboard occluder and stickers as rewards. Cups were placed upside-down on the shelves. On each set of shelves, there was one target cup in which a sticker could be hidden (left set: pink cup; right set: green cup), all other cups were distractor cups and were sealed at the bottom. See the electronic supplementary material, table S1 for details.

#### (i) Procedure

The task was split across 2 days to avoid fatigue (day 1: training 1 and 2; day 2: recap and test). On two further days, we administered three other attentional set shifting tasks for exploratory purposes (see the electronic supplementary material).

***Training 1.*** One target cup and one distractor cup were placed on each set of shelves. On the green set: green cup (target) and orange cup (distractor); on the bottom shelf on the blue set: pink cup (target) and yellow cup (distractor) on the top shelf (for photos, see the electronic supplementary material, table S1). The purpose of this phase was to familiarize children with the rules of the game: that only one specific cup was predictive of the sticker on each shelf and that children would switch between sets. See the electronic supplementary material for a description of all phases and a video uploaded to the OSF website for short clips of the training and test.

***Training 2.*** If children reached the learning criterion of training 1, they immediately continued with training 2. Two further distractor cups were added on each set of shelves (electronic supplementary material, table S1): On the green set, a blue and a red cup were placed on the upper shelf; on the blue set, a red and a blue cup were placed on the lower shelf. The purpose of this phase was to familiarize children with the fact that there were four cups on each set of shelves and to continue learning that on each set only one cup was rewarded and that the two sets of shelves would be presented in turns.

***Training 2 recap.*** The second day started with another training identical to training 2, used as warm-up. This was necessary because for some children the second research day was not the day immediately after the first owing to the weekend, illnesses or the nursery schedule.

***Test.*** In the test, the blue and red distractor cups were replaced by new distractors, so that now the same four cups were used on both sets of shelves: a green and an orange cup on the bottom shelf, and a pink and a yellow cup on the top shelf (electronic supplementary material, table S1 and figure S1). Over trials, cups changed their left–right position semi-randomly but stayed on the same shelf, so the arrangement of the cups was not necessarily the same on both sets of shelves. In order to select the correct cup, children needed to keep in mind which set they were currently presented with (green/right or blue/left). Children started the test with the set that they did not end with in the preceding training. Children were tested on one set until they chose correctly for three consecutive trials and then switched to the other set. Unlike in training, children did not receive feedback about the sticker location if they chose incorrectly. Children completed one test session with 36 trials. The test stopped once children completed 36 trials, if children lost interest or asked to stop. In the latter case, the experimenter ended the session and resumed the task in the following session.

#### (ii) Scoring and analysis

We coded for each trial which cup was selected, whether it was the correct choice and—in case of an unsuccessful trial—which of the three potential errors participants made: (i) selecting the correct shelf, but the wrong cup, (ii) selecting the wrong shelf and the cup that is relevant on the other set of shelves (switching mistake), or (iii) selecting the wrong shelf and the type of cup that has never been rewarded before (electronic supplementary material, figure S1). The latter two errors will be referred to as ‘wrong shelf errors’.

For the test, we scored the proportion of achieved switches out of all switches possible if the participant always chose correctly and thus switched after every three trials (see the electronic supplementary material for details), the number of errors directly after a switch (here we counted the first trial in the test as a ‘trial after a switch’, as children switched sets of shelves when they proceeded from the training to the test) and the proportion of errors directly after a switch which were coded as switching mistakes. See the electronic supplementary material, for a complete description of variables and analyses.

### (c) Results

#### (i) Training

Of the 46 children who started the task, one failed to meet criterion in the training and two children stopped as they lost interest. Age had a significant effect on performance only in training 2, with older children being administered significantly fewer trials than younger children (electronic supplementary material, table S2).

#### (ii) Test

Of the 43 children who continued to the test, five stopped it before the end of the task (after 1, 16, 19, 20 and 24 trials, respectively) and were removed from further analysis. The remaining 38 children completed all 36 test trials (electronic supplementary material, table S2). The mean proportion of errors out of all trials was 0.39 ± 0.25 (range 0–1). The mean proportion of achieved switches out of all possible switches was 0.53 ± 0.29, (range 0–1; electronic supplementary material, figures S2, S3a and S4a). Children's switching performance was significantly above chance level (0.22; two-sided Wilcoxon test, T + = 699, *p* < 0.001). The chance level was based on 100 000 Monte–Carlo simulations assuming random sampling of one out of two cups (representing sampling one out of the two previously rewarded cup types, not out of all four possible cups in a set), with 0.22 (s.d. = 0.12) being the proportion (and s.d.) of achieved switches assuming random sampling (see the electronic supplementary material, Scoring and Analysis). We deemed this to be a conservative and more realistic benchmark level for comparison, given that it was likely that participants who had passed the training stages and accumulated experience with the rewarded and non-rewarded types of cups would be more likely to randomly pick between the two previously rewarded cup types than to choose equally between the four cups, including the two that had never been rewarded before.

*Age effects.* While age did not significantly predict the proportion of achieved switches nor the proportion of switching mistakes out of all errors (see the electronic supplementary material), it was a significant predictor of the proportion of trials directly after a switch that were incorrect (−0.10 ± 0.03, *z* = −3.00, *p* = 0.003; electronic supplementary material, figure S7a and table S5), with older children making fewer errors in trials directly after a switch. With every month increase in age, the odds of making an error after a switch decreased by 10%.

#### (iii) Error patterns

*Switching errors.* Looking only at those children who made at least one error (all but one child, so *n* = 37), the proportion of switching mistakes out of the total number of errors was 0.78 ± 0.16 (range 50–1; electronic supplementary material, figure S9a), demonstrating that the majority of children's errors were specific switching mistakes.

*Errors after a switch.* The proportion of errors directly after a switch was 0.64 ± 0.31 (range = 0–1). The proportion of trials directly after a switch that were coded as specific switching mistakes was 0.63 ± 0.31 (range = 0–1), demonstrating that if a mistake was made after a shift, in 98% of cases, it was a switching mistake.

*Comparing the effect of conflicting and neutral distractors.* We compared the proportion of wrong shelf errors (i.e. either making a switching mistake or selecting the distractor cup on the wrong shelf) between trainings 2, 2 recap and the test, to explore the effect that introducing conflict in the test had on performance. Wrong shelf errors out of *all trials* occurred less often in training 2 recap (0.05 ± 0.09, range 0–0.5) than in training 2, (17 ± 0.08, range 0–0.35), paired-samples, two-sided Wilcoxon test, *V* = 730.5, *p* < 0.001; electronic supplementary material, figure S10), indicating that by training 2 recap, children had learned that the distractor cups were never rewarded (a similar pattern was found for the mean proportion of wrong shelf errors out of *all errors*; see the electronic supplementary material). In training 2, in which the distractor cups were introduced, all but one child chose one of the distractor cups at least once; the tendency to choose a distractor cup was among the most pronounced in the first trial (electronic supplementary material, figure S11). In the test, the proportion of wrong shelf errors increased again 0.35 ± 0.23 (range 0–1) and persisted across trials.

### (d) Discussion

E1 demonstrated that the task was suitable for children between 3 and 5 years: most children (95%) passed the training stages and 84% of children who started the test completed the 36 test trials. There were no floor nor ceiling effects—even the youngest children understood the task but 5-year olds did not find the game trivial either. On average, children managed to achieve about half of the possible switches in the test, and variance in performance was well spread (electronic supplementary material, figure S4).

We established content validity of the task: most errors (78%) were switching mistakes, i.e. mistakes in which children chose the cup that was predictive of the reward on the other shelf. That is, when children made errors, most of the time these resulted from children failing to re-direct their attention rather than from picking a distractor cup owing to general inattentiveness or guessing.

We investigated possible age effects (for comparability with E2), but as the age range in E1 was narrow (mostly 3- and 4-year olds, only one 5-year old), we treat these results with caution. In training 2, older children were administered significantly fewer trials than younger ones. In the test, older children were less likely to make an error in trials after a switch than younger children. There was no age effect on the proportion of achieved switches, the proportion of switching mistakes of all errors or on the likelihood of success on a trial-by-trial basis.

## 3. Experiment 2 (children)

E2 was part of a larger test battery [33], in which children aged 3 to 6 years were tested on a series of EF tasks. Each task was administered on a separate day, with the shifting shelf task being administered on days 3 and 4. Here, we only report the results from the shifting shelf task, with the aim to examine children's performance based on a larger sample and to examine the developmental trajectory of the task.

### (a) Methods

Materials, procedure, scoring and analysis were the same as in E1.

#### (i) Participants

We tested 178 children between 3 years 6 months and 6 years 0 months (mean = 49.91, s.d. = 6.95 months) in 18 nurseries and schools recruited in small and medium-sized cities in Scotland, UK, between June 2019 and March 2020. There were sixty-eight 3-year olds, seventy-seven 4-year olds, eleven 5-year olds, and one 6-year old (see the electronic supplementary material for information on ethnic and parental educational background). Owing to the SARS-CoV-2 outbreak, data collection stopped before all children had completed the test battery. Therefore, out of the 172 children who qualified for testing day 2, five children could not complete the task.

### (b) Results

#### (i) Training

As in E1, most children reached the learning criteria in the training. Of the 178 children who started training 1, 166 proceeded to the test. There were no significant age effects on performance. See the electronic supplementary material for details.

#### (ii) Test

The test was started by 166 children, but we had to remove some participants owing to experimenter error (9x) and children stopping the test before they completed 75% of the test trials (5x). Therefore, 152 children had valid data on the test. We included children who did not reach 36 trials but who had completed 75% of test trials (i.e. 27 trials or more): one child completed 31 trials, one 33 trials and seven 35 trials (these children lost trials owing to experimenter error) and one child stopped after 32 trials. Owing to experimenter error, we had to remove the first three trials for ID 55 and trial 21 for ID 139. For these children, we adjusted the maximum number of possible of switches.

The mean proportion of errors of all administered trials was 0.33 ± 0.20 (range 0–1; E1: 0.39), so similar to E1. The mean proportion of achieved switches out of the possible number of switches was 0.57 ± 0.26 (range 0–1; electronic supplementary material, figures S3b and S13), i.e. was slightly higher than in E1 (0.48), possibly owing to the inclusion of older children. Children's switching performance was significantly above chance level (0.22), T + = 11425, *p* < 0.001.

*Age effects on performance.* Age had a significant effect on all dependent variables. In contrast with E1, age in months had a significant effect on the proportion of achieved switches (estimate ± s.e.: 0.05 ± 0.01, *z* = 4.01, *p* < 0.001; electronic supplementary material, figure S5b and table S6), with older children achieving significantly more switches than younger children. Age also had an effect on the proportion of switching mistakes out of all errors (0.03 ± 0.01, *z* = 2.58, *p* = 0.010; electronic supplementary material, figure S6b and table S7), with older children having a lower proportion of making other, i.e. non-switching, mistakes than younger children. As in E1, there was a significant negative effect of age on the proportion of incorrect trials directly after a switch (−0.042 ± 0.014, *z* = −2.935, *p* = 0.003; electronic supplementary material, figure S7b and table S8).

#### (iii) Error patterns

*Switching errors.* Looking only at those children who made at least one error (all but four children, so *n* = 148), the proportion of switching mistakes out of the total number of errors was 0.76 ± 0.19 (range 0.21–1; electronic supplementary material, figure S9b). This again showed that most of children's errors were switching mistakes (and, as we showed above, with increasing age children made relatively more switching mistakes than random errors).

*Errors after a switch.* The proportion of incorrect trials directly after a switch was 0.57 ± 0.31 (range = 0–1), i.e. in over half of the trials directly following a switch a mistake was made (as in E1, older children were less likely to make an error after a switch than younger children). The proportion of trials directly after a switch that were coded as a switching mistake was 0.52 ± 0.32 (range = 0–1). Thus, as in E1, almost all mistakes made in trials after a switch were switching mistakes (91%).

*Comparing the effect of conflicting and neutral distractors.* As in E1, the proportion of wrong shelf mistakes decreased from training 2 to 2 recap, but in the test, these mistakes constituted the majority of errors—because the distractor cup on the wrong shelf captured children's attention as this type of cup was the correct option on the other set of shelves (electronic supplementary material, figure S11). This pattern was the same for all age groups (electronic supplementary material, figure S14). The mean proportion of wrong shelf errors out of *all trials* in training 2 was 0.21 ± 0.10 (range 0–1; E1: 0.17), in training 2 recap 0.07 ± 0.09 (range 0–0.31; E1: 0.05) and in the test 0.29 ± 0.18 (range 0–1; E1: 0.35), and thus very similar to the pattern observed in E1.

### (c) Discussion

Using a larger sample size and wider age rage than E1, we confirmed content validity by showing that three quarters of errors in the test were switching mistakes. Further evidence that errors were owing to stimulus conflict were found by comparing error patterns in the test phase with those in the training. In trainings 2 and 2 recap, children of all age groups showed a similar error pattern, in that they tended to make wrong shelf errors early on in training 2 (as they were exploring the newly added cups), a tendency that decreased in training 2 recap, in which fewer errors were made overall. In the test, the proportion of wrong shelf errors increased and persisted over trials for all age groups, indicating that the introduction of the conflict between the two potentially rewarding cups put a cognitive load on children.

There was no age effect in the training, indicating that children of all age groups learned the rules equally well. Yet, in the test, older children on average performed better than younger children, achieving a significantly higher number of switches and being significantly less likely to commit errors in trials directly after a switch. Older children had a higher proportion of switching mistakes out of all errors than younger children, indicating that younger children made more of the other types of errors. While children tended to perform better with increasing age, there was still sufficient variation in performance in the older age group. Thus, our task revealed age-related increases in children's attention shifting abilities, but continued to capture individual differences at each age group.

## 4. Experiment 3 (chimpanzees)

### (a) Methods

This experiment was also part of the test battery reported in [33]. It was administered as the last task, on testing days 16 (training 1), 17 (training 2) and 18 (test). Note that in contrast with the children, chimpanzees were only administered a single session of training 2 because for them training 2 could always be administered one day after training 1, whereas for children sessions were often separated by several days (owing to weekends, absences, testing schedule) and thus a warm-up session was deemed necessary for the children.

#### (i) Participants

We tested 53 chimpanzees from two sanctuaries (28 chimpanzees at Sweetwaters Chimpanzee Sanctuary, Ol Pejeta Conservancy, Kenya; and 25 chimpanzees at Ngamba Island Chimpanzee Sanctuary, Uganda), aged 5–36 years (median age: 21 years; for details see the electronic supplementary material). Chimpanzees were individually tested but were separated from the others for no longer than 30 min. There was only one test session per day and individual. The chimpanzees were fed multiple times a day with fresh fruits and vegetables and the research did not interfere with the feeding schedules.

#### (ii) Procedure

The procedure was largely the same as for the children. The two main differences were that there was only a single session of training 2 for the chimpanzees and that the maximum number of trials per training phase was 72, with a maximum of 108 trials to complete both phases. There were three testing sessions (one session per day): day 1, training 1; day 2, training 2; day 3, test.

### (b) Materials

The shelves were similar to the ones used with children (see the electronic supplementary material, figure S15). Banana pieces were used as a reward.

#### (i) Scoring and analysis

Scoring and analysis was the same as for children. We used beta regressions to compare performance between species (data for children were pooled across E1 and E2) and, for those variables for which we investigated and found age effects in children (i.e. proportion of achieved switches, proportion of switching mistakes out of all errors, proportion of trials directly after a switch that were coded as errors) we also compared performance between chimpanzees and the three age groups of children (3, 4 and 5 years).

### (c) Results

#### (i) Training

Of the 53 chimpanzees who started training 1, fifty qualified for the test phase (electronic supplementary material, table S2).

#### (ii) Test (and comparison to child samples)

Chimpanzees’ mean proportion of errors out of all administered trials was 0.37 ± 0.17 (range 0.08–0.80; E1: 0.39, E2: 0.33), which was within the range of the child data. The mean proportion of achieved switches out of the possible number of switches was 0.52 ± 0.21 (range 0.09–0.91; electronic supplementary material, figure S4; E1: 0.48, E2: 0.57) which was significantly above chance level (0.22), *t*_49_ = 9.86, *p* < 0.001. We compared the proportion of switches made by chimpanzees and the three age groups of children across E1 and E2 (note: the one 6-year-old child was added to the group of 5-year olds) using beta regression (electronic supplementary material, table S9). On average, 5-year olds achieved a significantly greater proportion of switches (0.80 ± 0.19, range 0.45–1) than chimpanzees (0.52 ± 0.21, range 0.09–0.91, *p* < 0.001), 3-year olds (0.50 ± 0.25, range 0.09–1, *p* < 0.001) and 4-year olds (0.59 ± 0.26, range 0–1, *p* = 0.007; electronic supplementary material, table S9). No other comparisons were statistically significant.

#### (iii) Error patterns (and comparison to child samples)

*Switching mistakes.* The proportion of trials in which a switching mistake was made was 0.26 ± 0.15 (range 0.03–0.58) and thus similar to children's performance (E1: 0.28, E2: 0.23). The proportion of switching mistakes out of *all errors* was 0.68 ± 0.23 (range 0.07–1; electronic supplementary material, figure S9c), and significantly lower than in children (E1: 0.78, E2: 0.76; beta regression: −0.412 ± 0.154, *z* = −2.67, *p* = 0.007). Split by age groups, the proportion of switching mistakes out of all errors was significantly lower in chimpanzees than 5-year olds (0.87 ± 0.13, range 0.73–1; *p* = 0.002) and 4-year olds (0.77 ± 0.18, range 0.35–1; *p* = 0.006), but not 3-year olds (0.73 ± 0.19, range 0.21–1, *p* = 0.151), nor were there differences between age groups (for estimates see the electronic supplementary material, table S10). Thus, when mistakes were made, older children (but not 3-year olds) were more likely than chimpanzees to commit specific switching mistakes.

*Errors after a switch.* The proportion of incorrect trials directly after a switch was 0.52 ± 0.31 (range = 0–1), i.e. in about half of the trials directly following a switch a mistake was made. While this proportion was slightly lower than what was observed in the children (E1: 0.64, E2: 0.57), the difference between species was not statistically significant (neither when averaged across age groups nor separate by age groups; see the electronic supplementary material for estimates). The proportion of trials directly after a switch that were coded as switching mistakes was 0.45 ± 0.33 (range = 0–1). This was lower than the proportions observed in the children (E1: 0.63, E2: 0.52), but again the difference was not statistically significant (see the electronic supplementary material). Thus, as for the children, the majority of incorrect trials after a switch were switching mistakes (86%). Finally, the proportion of incorrect trials that occurred after a switch was 0.23 ± 0.16 (range 0–0.8), which was significantly lower compared to the children (E1: 0.35, E2: 0.40; beta regression: −0.58 ± 0.17, *z* = −3.49, *p* < 0.001). That is, while for children more than a third of the errors occurred in trials directly after a switch, chimpanzees' errors seemed to occur slightly more independently of switches.

*Comparing the effect of conflicting and neutral distractors.* The mean proportion of wrong shelf mistakes out of *all trials* in training 2 was 0.14 ± 0.10 (range 0–0.51; E1: 0.17, E2: 0.21), and in the test 0.30 ± 0.17 (range 0.05–0.69; E1: 0.35, E2: 0.29), and thus in the range of the results for the children (electronic supplementary material, figure S10). As children, chimpanzees reached towards the wrong shelf more often in the test compared to the training as a result of the introduction of the conflict.

The mean proportion of wrong shelf errors out of *all errors* in training 2 was 0.48 ± 0.31 (range 0–1), which was significantly lower than the proportions for children (E1: 0.68, E2: 0.80), beta regression: −1.16 ± 0.19, *z* = −6.06, *p* < 0.001. In the test, it was 0.78 ± 0.20 (range 0.13–1; electronic supplementary material, figure S9), which was again significantly lower than for the children (E1: 0.92, E2: 0.90), beta regression: −0.65 ± 0.14, *z* = −4.61, *p* < 0.001. This suggests that: (i) analogous to what was found with the children, wrong shelf mistakes increased from training 2 to the test and remained high throughout the test (electronic supplementary material, figure S11); but that (ii) in contrast with the children, chimpanzees’ proportion of wrong shelf errors of all errors was significantly lower, demonstrating that chimpanzees committed a larger proportion of non-specific, ‘random’ errors.

### (d) Discussion

The shifting shelf task was suitable for chimpanzees. Only two chimpanzees dropped out of the training and all but one reached the training criteria. There were no floor or ceiling effects, and variance was well spread (electronic supplementary material, figure S4c). Chimpanzees, 3- and 4-year-old children achieved a comparable proportion of switches (50–59% of possible switches), but these groups were outperformed by 5-year olds (80%).

As found for children, the majority of chimpanzees' mistakes were switching mistakes (68%). This proportion was comparable to the level of 3-year olds (73%), but significantly lower than in 4-year olds (77%) and 5-year olds (87%). In about half of the trials directly after a switch chimpanzees made an error (52%), which was not statistically different from 3-year olds (64%), 4-year olds (59%) nor 5-year olds (26%; this comparison was marginally significant but note the low sample size of 5-year olds (*n* = 13)).

As observed with the children, wrong shelf errors increased from the training to the test, indicating the additional demand of attention shifting. Yet, in both phases the proportion of wrong shelf errors of all errors was lower than what was observed for children, showing that chimpanzees made a greater proportion of non-specific errors than children (i.e. choosing the cup next to the rewarded cup).

## 5. General discussion

We add to the literature a non-verbal attentional set shifting task suitable for preschoolers and non-human primates. To establish content validity, we created a task eliciting a specific error pattern [50]: switching mistakes. The task is suitable for children and chimpanzees, showing low drop-out rates, a broad spread in performance variance, and capturing a clear signature of attention shifting as errors were primarily switching errors, i.e. mistakes that resulted from a failure to switch between two mental sets or rules.

We found evidence for performance improvement over the preschool years: older children made fewer mistakes than younger children and were less likely to make a mistake directly after a switch (i.e. when the need for attentional set shifting is greatest). If mistakes were made, older children were more likely to make switching mistakes (indicating a failure to switch attention), whereas younger children were more prone to choosing a cup that has never been rewarded. This might suggest that older children had formed a stronger attentional set during the training compared to the younger children. This would be in line with the *Selective Attention Theory* proposing developmental changes throughout the preschool years in how children exert selective attention ([51–53]; see also [17]): older preschoolers have a greater capacity than younger children to focus and maintain their attention on the relevant stimulus/dimension (in an ‘all-or-none’ fashion). While this results in older children making fewer mistakes overall, if they make a mistake they would be expected to choose the only other type of cup on the shelf that has received a heavy attentional weight in preceding trials, namely the cup that is rewarded on the other shelf. By contrast, for younger children, the difference in weights between the cups would be more graded and thus they would be expected to choose more often one of the cups that have never been rewarded.

Regarding species comparisons, we found three main results. Chimpanzees (52%), 3-year olds (50%) and 4-year olds (59%) achieved a comparable proportion of switches in the test, whereas 5-year olds (80%) significantly outperformed these groups. While in all these groups, the majority of errors were switching mistakes (indicating a failure to switch attention), the proportion of switching mistakes in chimpanzees (68%) was significantly lower in 5-year olds (87%) and 4-year olds (77%; but not 3-year olds (73%)), suggesting that chimpanzees had a somewhat higher rate of making ‘random’ errors than older children. When looking at trials directly following a switch (i.e. the trials in which the demand on attention shifting is highest), we found that in a (slight) majority of them an error occurred (chimpanzees (52%), 3-year olds (64%), 4-year olds (59%), 5-year olds (57%); see the electronic supplementary material, table S2). While there were no statistically significant differences between groups, there was a marginal significant trend for 5-year olds fitting the above pattern (note the low sample size for 5-year olds, *n* = 13). A future study with a greater number of 5-year-old children could investigate this age trend further. These results suggest that the attentional set shifting abilities in this task are on a comparable level between 3-year-old preschool children and chimpanzees, but that there might be quantitative and qualitative changes in this skill in humans from 5 years of age. The strengths of the attentional set formed by the chimpanzees in this study seemed to be similar to the one of younger children, but weaker than the attentional set formed by the 5-year olds. This interpretation is in line with Pope *et al*. [43] who found that chimpanzees and baboons were less susceptible to forming a cognitive set compared to humans in the initial learning phase of a sequence learning touchscreen task. Underpinning this development is probably an interplay of ‘biological’ and ‘cultural’ factors that boost children's attentional set shifting abilities, such as the biological maturation of fronto-parietal brain regions, allowing for greater capacities of other core cognitive abilities such as inhibitory control and working memory, and the acquisition and use of cultural tools such as language, rules, values, knowledge and beliefs that facilitate sustaining and switching attention to stimuli [54–58].

The results of this study fit with the findings of Herrmann & Tomasello [21] showing that attention shifting in 5-year olds was significantly better than in chimpanzees in a task in which participants had to monitor and switch attention between two apparatuses, whereas 4-year olds and chimpanzees performed on a comparable level. However, an important difference between this and the current study is that Herrmann & Tomasello's [21] task did not include the need for solving a cognitive conflict that can only be solved by applying and switching between rules (see e.g. the DCCS or ‘no-change’ versions of the ID/ED paradigm). The findings also fit with the results from an unpublished PhD thesis [59] in which chimpanzees, 4- and 5-year-old children, and adults were tested in an ID/ED task (*shifting boxes* task) in which they were presented with pairs of boxes, differing in shape and filling material (see also [33]). In the pre-switch phase, participants could learn that a specific *filling material* was predictive of the reward. In the critical ED phase, the rule changed (unannounced), with one of the *shape features* becoming predictive of the reward. Again, chimpanzees and 4-year olds performed on a comparable level, while being outperformed by 5-year olds (but still half of the 5-year olds failed to switch attention), with human adults outperforming all groups.

We see three main limitations to this study. First, data on the test–retest reliability of the shifting shelf task are lacking (note that results on split-half reliability are reported in the electronic supplementary material, p. 44). Assessing test–retest reliability is an important next step in relation to this task, in order to examine whether it is not only able to capture group-level differences (as shown here), but also potentially suitable as a measure of stable individual differences [60]. Second, while we were able to show elsewhere [33] that children's (but not chimpanzees') performance in the shifting shelf task was significantly correlated to another new attentional set shifting task (the shifting tray task), we do not have (sufficient) data on how performance on the shifting shelf task compares with performance on other, already established attentional set shifting tasks. While we also collected data on a virtual version of the DCCS on a tablet device in E1, the final sample size of children who participated in both tasks resulted in insufficient statistical power for correlational analyses. Third, the findings regarding the children are limited to a population of Westernized, mainly White, children of relatively highly educated parents. Data from more diverse socioeconomic and cultural backgrounds are needed to assess generalizability of the current findings and to help us to better understand the social, cognitive and contextual factors underpinning cognitive flexibility.

To better understand differences and commonalities in attentional control, set shifting and EF generally between humans and non-human primates, we need studies using batteries of tasks (and thus also more tasks suitable for comparative research), to allow for better generalization across task idiosyncrasies. To inform our understanding of the evolution of attentional control in general, we also need studies on non-primate species. Owing to its non-verbal nature, the shifting shelf task should be easily adaptable for use with different animals and groups of humans. It can also be easily scaled up or down, and the alternating presentation of the sets of shelves could be automated. The task is adaptable to an animal's object manipulation abilities: depending on whether the animal has arms, a beak, trunk or uses its mouth, the cups can be modified to allow easier manipulation (e.g. by adding handles, using boxes with lids or drawers instead of upside-down cups). More importantly, the task does not require any interaction with the materials, as long as the animal can make a choice by pointing (as done with the chimpanzees in the current study) or pecking, without having to open the cups themselves. A translation of the task into a virtual touchscreen task is also possible and would allow for greater automatization, the measurement of reaction times and possibly an easier modification of the difficulty level (e.g. by modifying the context cue indicting which cup is the correct one—e.g. instead of using the left–right location and different colours for the shelves, a single, more subtle visual cue could be given). Finally, the task could be used to assess the impact of socially learned tools such as verbal labels on attentional set shifting, by testing non-human primates of different degrees of enculturation (including language training), as well as evaluating the relationship between learning labels and performance in children [61].

## Data Availability

Data and analysis code available at https://osf.io/c9sdh/?view_only=1bf2cf7109604557a3d0186b10334cbd (doi:10.17605/OSF.IO/C9SDH). The data are provided in the electronic supplementary material [62].
